# Feasibility and Preliminary Effectiveness of a Health and Wellbeing Intervention for Adults With Mild to Moderate Intellectual Disabilities

**DOI:** 10.1111/jar.70320

**Published:** 2026-10-19

**Authors:** Helen McCready, Ken van Someren, Ben Fitzpatrick

**Affiliations:** ^1^ Atlantic Technological University Donegal Ireland; ^2^ University of Ulster Derry/Londonderry UK

**Keywords:** acceptability, feasibility, fidelity, health, implementation framework, intellectual disabilities, MRC, physical activity

## Abstract

**Background:**

Many adults with intellectual disabilities attend day services, yet few intervention studies report implementability and preliminary effectiveness within day services.

**Methods:**

Guided by the Medical Research Council framework, this study evaluated early‐stage feasibility of an 8‐week health and wellbeing intervention for adults with mild and moderate intellectual disabilities (*n* = 24). Feasibility, acceptability, and fidelity were assessed alongside pre‐post outcomes, with exploratory analyses by intellectual disability level.

**Results:**

Retention was 100% and compliance was 73%. Outcome completion exceeded 80% for most measures, no adverse events occurred, and fidelity was satisfactory. Systolic blood pressure, muscular endurance and flexibility improved. Exploratory analyses suggested more favourable outcomes for participants with mild intellectual disabilities.

**Conclusion:**

The intervention was feasible and acceptable, with satisfactory fidelity, supporting progression with refinement. Preliminary health improvements were observed.

## Lay Summary

- People with intellectual disabilities are often in poorer health and less likely to take part in research that aims to improve health and wellbeing.
- There is very little research on health programmes delivered in intellectual disabilities day‐service settings.
- This study tested an 8‐week health and wellbeing programme for adults with intellectual disabilities in a day‐service setting in the community.
- The programme was safe to run, and people were willing and able to take part.
- Blood pressure, leg strength, and flexibility improved.
- People with mild intellectual disabilities improved their handgrip strength and balance.
- Personal wellbeing scores did not improve.
- The programme only needs small improvements before carrying out a larger study.

## 1 Background

People with intellectual disabilities experience substantial and persistent health disparities compared to the general population, including higher rates of multi‐morbidity and hospital admissions (Kinnear et al. 2018). Individuals with intellectual disabilities also have a higher risk of cardiovascular disease, in particular ischaemic stroke and death due to circulatory diseases (Cho et al. 2025). In addition, strength, balance, and flexibility are important for supporting daily functioning and participation. Difficulties in these physical attributes have been associated with increased fall risk, additional disability, and reduced engagement in daily and leisure activities (Lahtinen et al. 2007).

A growing body of evidence suggests that structured exercise interventions, including aerobic, resistance, and balance training, can improve physical fitness and cardiovascular health in adults with intellectual disabilities (Obrusnikova et al. 2022; Carmeli et al. 2005; Oviedo et al. 2014; Tsimaras and Fotiadou 2004). However, the evidence remains inconsistent, with substantial variation in methodological quality, intervention design, and outcome measures utilised (Greene et al. 2025; Jacob et al. 2023; St. John et al. 2020; Willems et al. 2018; Obrusnikova et al. 2022), and more research into effective exercise components for intellectual disabilities populations is welcomed (American College of Sports Medicine 2022; Salomon et al. 2023). Research impact is limited by poor sustainability, as interventions are often not adequately adapted for people with intellectual disabilities due to the lack of consideration for social support systems and environmental barriers, and long‐term follow‐up studies are lacking (Willems et al. 2018). The intervention setting may serve as an enabler or barrier of intervention success. Delivering lifestyle modification programmes in the routine daily context where support systems are in place, and with respect for usual routines, may enable engagement (Hassan et al. 2019). Review has shown that where established routines of adults with intellectual disabilities are not respected during implementation, distress and lower motivation to participate can result (Westrop et al. 2024). Respecting the day‐to‐day routines as a predictable foundation can provide adults with intellectual disabilities with a base from which to build capabilities, and utilising the support systems available, active participation is positively influenced (Elinder et al. 2018; Westrop et al. 2024).

Physical activity has been linked to psychological wellbeing, but appropriate and reliable measurement tools for individuals with intellectual disabilities remain limited (Bondár et al. 2020). While validated scales exist such as the Glasgow Depression Scale for people with a Learning Disability (Cuthill et al. 2003) and the Personal Wellbeing Index—Intellectual Disabilities (Cummins and Lau 2010), feasibility and acceptability concerns persist as participants may have difficulty answering questionnaires, even with support (Lally et al. 2022). Where measures rely on abstract or introspective concepts (mood, wellbeing, life satisfaction), as reflected in commonly used tools, such response challenges may arise (Melville et al. 2015). Self‐report ratings are typically higher than proxy ratings across all quality‐of‐life domains (Koch et al. 2015), raising questions about the validity and feasibility of measurement approaches.

Given that many adults with intellectual disabilities access day services (Inclusion Ireland 2021), it is important to understand whether interventions can be delivered effectively within these routine settings. This shifts the focus from effectiveness alone to questions of feasibility, acceptability, and implementability of planned interventions for the participants within their usual day‐to‐day setting (Bellamy et al. 2020; Salomon et al. 2018).

The Medical Research Council (MRC) framework for developing and evaluating complex interventions emphasises the importance of early‐stage evaluation before large‐scale implementation or effectiveness testing (Skivington et al. 2021). Early‐stage evaluations help determine whether an intervention warrants progression, refinement, or replacement (Atkins and Michie 2015; Skivington et al. 2021).

Complementing the MRC framework, Klaic et al. (2022) identify feasibility, acceptability, and fidelity as core constructs for early‐stage evaluation. Feasibility refers to whether the intervention could be carried out with the intended population, acceptability to how suitable and satisfactory it is for participants, and fidelity assesses whether it is delivered as intended (Klaic et al. 2022). While definitions vary across studies, these constructs are widely recognised as central to early‐stage evaluation (Klaic et al. 2022; Sekhon et al. 2017).

Rana et al. (2024) highlight the need for population‐specific interventions with robust methodological reporting. One important, but underexplored factor, is level of intellectual disability, which may influence both intervention feasibility and outcomes (Rana et al. 2024). Existing reviews have shown that studies rarely report findings by level of intellectual disability (Shin and Park 2012; Westrop et al. 2019, 2024) but acknowledge that interventions must be delivered in ways that respect the cognitive and physical abilities, as well as support needs, of all participants as prior review has demonstrated (Westrop et al. 2024). Evidence consistently finds that increasing severity of intellectual disability is associated with decreasing levels of physical activity, with all identified studies in one review reporting this inverse relationship (Vancampfort et al. 2022). This is concerning given the evidence for increased mortality due to cardiovascular disease among individuals with more severe intellectual disabilities (O'Leary et al. 2018). In parallel, many outcome measures and feasibility metrics used within physical activity interventions have been validated primarily in people with mild to moderate intellectual disabilities (Enkelaar et al. 2013), with a marked absence of people with more severe levels, or with behaviours of concern (Greene et al. 2025). Consequently, the current evidence base is skewed, limiting the applicability to individuals with more complex support needs (Greene et al. 2025). Within studies, feasibility metrics or treatment responses of participants with mild or moderate levels of intellectual disabilities are continually presented as one unified grouping (Chow et al. 2018; Oviedo et al. 2014; Shields et al. 2013), with few exceptions (Guerra‐Balic et al. 2015). People with moderate intellectual disability may require more support, adaptations, prompts, demonstrations, and simplified materials than people with mild intellectual disabilities (Hartley and MacLean 2006), irrespective of the type of intervention and modifications should be made at an individual level (Piantedosi and O'Shea 2025). Reporting outcomes by distinct level of intellectual disabilities, and in particular feasibility outcomes may identify implementation issues before a definitive trial.

### 1.1 Study Aim

The primary aim of this study was to assess the feasibility, acceptability and fidelity of delivering and evaluating a community‐based health and wellbeing intervention for adults with mild and moderate intellectual disabilities, delivered within the existing day service routine.

The secondary aim was to examine the preliminary effectiveness of the intervention, with an exploratory subgroup analysis of how the level of intellectual disability (mild, moderate) influences feasibility, acceptability, and preliminary effectiveness.

The overall objective is to determine whether the intervention warrants progression to a full randomised control trial, or refinement or replacement before further pilot testing.

## 2 Methodology

This study used quantitative methods to assess the feasibility, acceptability, fidelity, and preliminary effectiveness of an existing intervention designed for community‐based application to determine if the intervention could progress under research conditions within the day service setting. This study followed the MRC framework and, as an early‐stage feasibility evaluation and as it was not a clinical trial of effectiveness, formal trial registration was not required.

### 2.1 Background to the Health and Wellbeing Programme

The Health and Wellbeing programme was an existing multi‐component programme promoting the physical health and personal wellbeing of adults with intellectual disabilities attending intellectual disabilities day services. It was designed and delivered by exercise scientists external to the day service and implemented in community settings, such as community and leisure centres. Guided by the MRC Framework, the programme met the criteria for a complex intervention as it involved multiple components, flexibility within components, and required skilled facilitation (Skivington et al. 2021).

The programme ran 4 days per week for 8 weeks, with morning and afternoon sessions. Participants attended during their usual day service hours, supported by day service staff. All programme content was facilitated by the Programme Facilitator. The day service staff were there for the support of the participants generally. Programme components, frequency, intensity, duration, and modality are summarised in Table 1, with a sample week provided in Table 2. All day service users arrived at their routine setting on the usual transport provided in the morning and remained with the group for their morning meeting and morning tea. After this, the group separated; those on the programme went to the community location of the programme, and non‐participants continued with the routine activities of the day service (e.g., in‐house literacy, communications, art, cookery, social outings). Lunch was between 12 and 1.30 pm, and during this time participants could either stay within the community for lunch or return to the day service centre, supported by the staff, who provided transport if needed. All community settings were a maximum of 15 min from the day service setting. Spaces were large, similar in size to a basketball court or community hall, with tables and chairs available. The afternoon session commenced at 1.30 and finished between 2.30 and 3, when participants returned to the day service for the afternoon tea before transport to their accommodation returned. Not accounted for within Tables 1 and 2 are ice breaker sessions, rest and recovery periods and transitions. Transitions included verbal and visual information on what to expect in the next session. Participants were not differentiated by level of intellectual disability and were familiar with one another through attending the day service.

**TABLE 1:** The health and wellbeing programme components.

Component	Critical component	Activity modality	Duration per session	Frequency
Physical exercise	Cardiorespiratory fitness training	Walking, dance, gym‐based Cardio machines, active games	45 min	3 times per week
	Strength training	Body weight exercises, Pilates, gym‐based weights machines	30 min	2 times per week
	Flexibility training	Yoga class	45 min	1 time per week
	Balance training	Tandem stance, Tandem walking, single‐leg stance games	15 min	1 time per week
Personal wellbeing	Mindfulness sessions	Guided meditation, body scan	15 min	2 times per week (end of day)
	Health education workshops: nutrition, healthy choices, emotional wellbeing	Worksheets, art and craft, role play and drama	30–60 min	2–3 times per week
Rest	For physical exercise every 10 min or earlier if needed by participant			
Intensity	Self‐selected by participant throughout physical exercise, monitored by the Talk Test (Reed and Pipe 2014, 2016)			

**TABLE 2:** Sample weekly schedule.

Session	Monday	Tuesday	Wednesday	Thursday
Session 1 (am)	CFT Walking in nature	Strength Strength class at the gym	CFT Active games at community centre (walking football, basketball)	CFT Dance class
Session 2 (am)	Health education (emotional resilience through self‐esteem bucket activity)			
	Lunch	Lunch	Lunch	Lunch
Session 3 (pm)	Flexibility (mat‐based session)	Health education (Nutrition: the food pyramid art exercise)	Health education (continue with Food pyramid: create meals)	Strength Pilates
Session 4 (pm)	Mindfulness (guided meditation)	Balance (Single leg balance games)	Mindfulness (body scan)	Review of the week

Abbreviation: CFT, cardiorespiratory fitness training.

#### 2.1.1 Programme Delivery

The programme was delivered across three cohorts, with an average of eight participants per cohort. The programme was delivered by exercise scientists who acted as programme facilitators. The lead facilitator delivered the intervention sessions and support facilitators were on site to train for consistency of delivery. This practice ensured that if the lead facilitator could not be present during the intervention, consistency of facilitation and delivery was ensured. Standardised protocols were agreed for the outcome measures utilised (Bellg et al. 2004). The lead facilitator also conducted baseline and post‐intervention outcome assessments. The principal investigator observed assessment procedures to evaluate adherence to the standardised protocols. The programme was never led by the day service staff, though many staff did get involved in activities. Staff accompanied participants to and from community venues, supported communication, encouraged participation, supervised breaks and transitions, and provided general participant support throughout programme delivery. Across cohorts, participant support was available at an approximate ratio of 2:1 participant to day service staff.

#### 2.1.2 Sampling and Recruitment

Inclusion criteria for participation in the health and wellbeing programme were aged ≥ 18 years, mild to moderate intellectual disability, physical ability to complete outcome measures, and meeting eligibility criteria of the pre‐testing screen for the Personal Wellbeing Index—Intellectual Disability [PWI‐ID] (Cummins and Lau 2010). Information on the level of intellectual disability was collected from information available through the day service. Individuals with mild intellectual disabilities can learn practical life skills, which allows them to function in ordinary life with minimal levels of support. Individuals with moderate intellectual disabilities can take care of themselves, travel to familiar places in their community, and learn basic skills related to safety and health and require moderate levels of support to self‐care (Patel et al. 2020).

Participants were recruited via convenience sampling from a pool of 47 adults attending a day service for adults with intellectual disabilities. All prospective participants received an information leaflet and were invited to an information workshop. From this pool, 40 prospective participants chose to attend the workshop, of which 13 people did not meet the inclusion criteria using the PWI‐ID pre‐screening questions (*n* = 9) or having physical incapacity (*n* = 4). Those that did not attend the workshop were not interviewed regarding their decision, and it was accepted as an execution of their autonomy and voluntariness. The remaining 27 participants provided informed consent and were enrolled in the study. Three enrolled participants did not commence the programme due to personal choice (*n* = 2) and ill‐health (*n* = 1), resulting in a final sample of 24 participants.

#### 2.1.3 Ethical Conduct

Empowerment of the participant to self‐consent was a principle of this study and procedures to ensure full ethical protections were followed. All data were anonymised, and physical assessments were conducted safely in appropriate settings.

#### 2.1.4 Consent

Easy‐read consent forms, verbal prompting, and an information workshop were used to support participants' understanding of the study. Consent forms were completed in a one‐to‐one meeting with each participant when comprehension was evident. Day service staff were present throughout, and verbal consent was reconfirmed before activities. During the consent process, the right to withdraw, at any time, was restated to participants.

#### 2.1.5 Participants

Demographic data were obtained from day service records for the 24 participants who commenced the programme (Table 3).

**TABLE 3:** Baseline characteristics of participants.

Variable	Baseline characteristics
Gender	*n* (% of sample)
Men	12 (50%)
Women	12 (50%)
Age: (mean ± S.D. years)	25 (±9)
ID level	*n* (% of sample)
Mild	9 (37.5%)
Moderate	15 (62.5%)
Type of ID	Unknown intellectual disability (*n* = 7) Intellectual disability + cerebral Palsy (*n* = 2) Autism spectrum disorder + moderate intellectual disability (*n* = 6) Down syndrome + mild intellectual disability (*n* = 1) Down syndrome + moderate intellectual disability (*n* = 6) Tetrasomy X syndrome + mild intellectual disability (*n* = 1) Smith Magenis syndrome + moderate intellectual disability (*n* = 1)

Abbreviation: ID, intellectual disability.

#### 2.1.6 Testing Procedures

Time was spent with the participants prior to testing for familiarisation with individual needs and communication strategies. Participants attended one to two familiarisation sessions before completing each physical outcome measure at baseline and post‐intervention. Testing occurred over two mornings at baseline and two mornings post‐intervention, with anthropometric measures collected first, followed by physical health and wellbeing assessments. Outcome assessments were conducted in line with pre‐agreed protocols standardised assessment procedures. Day service staff were present throughout to support participants and facilitate communication.

### 2.2 Data Collection Methods

#### 2.2.1 Feasibility, Acceptability and Fidelity Outcomes

Feasibility was investigated directly using recruitment rate, programme compliance, programme completion, and outcome measure completion. Recruitment rate was calculated as the proportion of the sampling pool meeting inclusion criteria who commenced the programme. Programme compliance was measured as the percentage of total sessions attended, programme completion as the total number of participants that started and finished the programme. Outcome measure completion was the proportion of participants completing assessments at both baseline and post‐intervention. Feasibility also related to the presence of acquiescent responding by identifying respondents that scored the PWI‐ID consistently at the maximum or minimum across all domains.

Acceptability was explored using a missing data analysis and the absence of adverse events was used as an indirect indicator of acceptability of procedures. Adverse events were any incidents occurring due to programme activities that led to withdrawal or time‐out from the programme (new injuries, exacerbations of pre‐existing conditions).

Daily logs were employed to assess the fidelity of the programme. Content fidelity of the intervention was measured using a weekly log of the core components delivered and examined for correspondence to those planned (Table 1). Fidelity of assessment procedures was evaluated through observational analysis of the assessors' consistency with the standardised assessment protocols that were established prior to data collection.

#### 2.2.2 Thresholds for Progression

With guidance from the CONSORT extension for pilot and feasibility trials (Eldridge et al. 2016), a priori thresholds for progression were agreed in line with the existing literature pertaining to recruitment, completion, adherence, adverse events and fidelity (Bellamy et al. 2020; Borrelli 2011; Conneely et al. 2024; Crowe et al. 2022; Denne et al. 2025). The literature shows that recruitment rates generally rest at 50% or higher, and rates lower than 30% are deemed unacceptable for progression (Bellamy et al. 2020; Conneely et al. 2024; Denne et al. 2025). Compliance required more stringent values, with rates greater than 70% feasible for progression and rates less than 50% considered unfeasible (Denne et al. 2025; Bellamy et al. 2020). Completion rates of outcome measures were less reported; however, a feasibility study involving a vulnerable population set a threshold of 80% for progression without amendment, and anything less than 60% was deemed unfeasible (Conneely et al. 2024). Whilst numerous studies report on the feasibility outcomes as described in this study, few report a priori thresholds for fidelity measures, despite guidance available (Borrelli 2011; Crowe et al. 2022). Following a synthesis of a priori progression threshold rates set, and results of feasibility papers published, consultation took place within the programme development team and the research team to agree on a priori thresholds for this study. To improve transparency and subsequent application, a traffic light system was used (Conneely et al. 2024; Denne et al. 2025), however, thresholds were considered a guide and support to decision making and not definitive (Eldridge et al. 2016). As this programme was not developed as part of this study, it was agreed that if initial feasibility, acceptability and fidelity was medium (amber) or low (red), based on a priori thresholds, a decision to refine (if medium/amber) or return to the development stage (if low/red) would be considered in consultation with the programme development team (Table 4).

**TABLE 4:** Feasibility, acceptability, fidelity and progression decisions.

	GO: feasible and proceed	AMEND: adjust the protocol, then proceed	STOP: trial and programme are not feasible
Recruitment rate	> 50%	30%–50%	< 30%
Programme completion rate	> 70%	50%–70%	< 50%
Programme compliance	> 80%	60%–80%	< 60%
Outcome measure completion	> 80%	60%–80%	< 60%
Adverse events	No events recorded	Event recorded – minor, not requiring medical intervention	Event recorded – led to hospitalisation due to injury
Content fidelity	High fidelity	Moderate fidelity	Low fidelity
	> 80%	51%–79%	≤ 50%

*Note:* Green denotes GO: Feasible and Proceed, amber denotes AMEND: Adjust the protocol, then Proceed, and red denotes STOP: Trial and programme are not feasible.

#### 2.2.3 Anthropometry

Body Mass Index (BMI) was calculated as weight (kg) divided by height (m^2^). Height was measured using a stadiometer (Seca 213, Hamburg, Germany) and weight using calibrated scales (Seca 813, Hamburg, Germany) following standardised procedures.

#### 2.2.4 Blood Pressure

Blood pressure was measured in the morning using an automated sphygmomanometer (Exact Fit 5 Connect, Braun) following recommended protocols (Daskalopoulou et al. 2012). The mean of the second and third readings from the left arm was used.

#### 2.2.5 Lower Body Muscular Endurance

Lower body muscular endurance was measured using the 30s Sit to Stand Test (Hilgenkamp et al. 2014). Participants performed as many complete sit‐to‐stand cycles as possible in 30 s, following familiarisation and demonstration.

#### 2.2.6 Isometric Handgrip Strength

Isometric handgrip strength was measured using a handheld dynamometer (Hydraulic Hand Dynamometer, Jamar; Netherlands) in a seated position. The mean of the strongest hand over three attempts was used.

#### 2.2.7 Flexibility

The *V*‐shape sit and reach test was used to measure flexibility of the hamstrings and lower back (Wu et al. 2010). The mean score of three trials was used.

#### 2.2.8 Balance

The Single Leg Balance Test was used to assess the static balance of participants. Modifications were made to standard protocols to suit the population. Hands could be used to assist balance and the clearance of the non‐tested foot off the floor was sufficient (Blomqvist et al. 2012). The time to failure was noted as when the non‐tested leg touched the floor and the best time over three trials was recorded from the best leg recorded (American College of Sports Medicine 2022).

#### 2.2.9 Cardiorespiratory Fitness

The 6‐min walk test (6MWT) was used to assess cardiorespiratory fitness following established guidelines (American Thoracic Society 2002), modified using practise trials, verbal encouragement, and pacing support as needed (Calders et al. 2011; Guerra‐Balic et al. 2015; Nasuti et al. 2013). The number of metres covered during 6 min was recorded.

#### 2.2.10 Personal Wellbeing

The Personal Wellbeing Index‐Intellectual Disabilities (PWI‐ID) assessed personal wellbeing. The PWI‐ID pre‐screening tool was used to assess suitability of application (McGillivray et al. 2009), and simplified scales were used as per the PWI‐ID operating manual (Cummins et al. 2010).

### 2.3 Data Analysis

Feasibility, acceptability and fidelity data are presented descriptively, as frequencies and percentages. Continuous data are reported as mean ± standard deviation (S.D.). Non‐continuous data is presented as frequencies (missing data and adverse events) and as percentages (recruitment, completion, compliance). All outcome variables were analysed for normality using the Shapiro–Wilk test alongside visual inspection of histograms and Q‐Q plots. Changes between baseline (Pre) and post‐intervention (Post) were analysed via paired samples *t*‐tests for outcomes with normally distributed difference scores, and Wilcoxon signed‐rank tests for outcomes with non‐normally distributed difference scores. An exploratory analysis using the procedure detailed above was conducted on the data to explore preliminary effectiveness by level of intellectual disability, with separate analysis of changes in the mild and moderate levels groups. Cohen's *d* values (*d*) were calculated for parametric data using the mean difference of pre‐ and post‐test scores divided by the standard deviation of the difference scores (Sullivan and Feinn 2012). In the case of non‐parametric data, the *r* value for effect sizes was calculated by dividing the *Z* value (standardised test statistic) by the square root of the *N* value (non‐tied pairs) (Tomczak and Tomczak 2014). Effect sizes were interpreted according to previous literature where low, medium and large effect sizes are represented by the cut‐off values < 0.3, 0.3 to ≤ 0.8 and > 0.8 respectively (Shin and Park 2012). All statistical analyses were conducted using IBM SPSS Statistics (v.29.0.1.0) with a *p* value of 0.05. During data analysis, responses to the PWI‐ID consistently at the maximum or minimum were excluded from analysis.

## 3 Results

### 3.1 Feasibility, Acceptability and Fidelity Outcomes

Informed consent was obtained from 27 of 47 adults with intellectual disabilities (57%) attending the day service. Twenty‐four participants commenced and completed the programme. Thirteen attendees of the information workshop did not meet the inclusion criteria. Programme session compliance was 73%, with absences due to holiday or illness. Physical health outcomes were fully completed except flexibility (2 refusals) and balance, where five participants (21%) could not perform the task, of whom four had moderate intellectual disabilities (Table 5).

**TABLE 5:** Feasibility, acceptability and fidelity results.

Outcome	Measure	(*n*)	%	Progression decision
Feasibility	Initial research pool	47		
	Expression of interest	40		
	Met Inclusion criteria and consented	27	89	
	Commenced programme	24		
	Recruitment rate		57	GO
	Programme compliance		73	GO
	Programme completion	24	100	GO
	Tests completed			
	Anthropometric	24	100	GO
	Blood pressure	24	100	GO
	Lower body muscular endurance	24	100	GO
	Isometric handgrip strength	24	100	GO
	Flexibility	22	92	GO
	Balance	19	79	AMEND
	Cardiorespiratory fitness	24	100	GO
	Personal wellbeing	16	67	AMEND
Acceptability	Measure	(*n*)		Progression decision
	Adverse events	0		GO
	Measure	(*n*)	Reason	Level of intellectual disability (*n*)
	Missing data analysis			
	Flexibility (sit and reach test)	2	Refused to sit on floor	Mild (1) Moderate (1)
	Balance (single leg balance test)	5	Unable to perform test	Mild (1) Moderate (4)
	Personal wellbeing (PWI‐ID)	8	Refused to retest	Mild (1)
			Ceiling effect detected	Mild (2) Moderate (5)
Fidelity	Measure			
	Content fidelity (core components)	Sessions planned (*n*)	Sessions delivered (*n*)	Delivered as percentage of planned (%)
	Cardiorespiratory	24	31	129
	Strength	16	15	94
	Flexibility	8	6	75
	Balance	8	5	63
	Mindfulness	16	9	56
	Health education	20	16	80
	Assessment fidelity	Satisfactory – all procedures followed.		

*Note:* Green indicates that the predefined ‘GO’ criterion was achieved; amber indicates the predefined ‘AMEND’ criterion; and red indicates the predefined ‘STOP’ criterion. For content fidelity, green indicates high fidelity (> 80%), amber indicates moderate fidelity (51‐79%), and red indicates low fidelity (≤ 50%).

Acceptability was supported by the absence of any adverse events. Missing data analysis found that participants completed most of the physical health outcomes measures above the acceptability rate of 80% set in the a priori thresholds for progression (Table 4). Inability to perform the balance test was recorded for five participants, most of whom have a moderate level of intellectual disability (*n* = 4). For personal wellbeing, PWI‐ID responses were excluded from data analysis (*n* = 7) because all domains were scored at the maximum value, indicating a possible acquiescent response pattern (Cummins and Lau 2010) and one participant declined the post‐intervention PWI‐ID (Table 5). The PWI‐ID and Single Leg Balance fell below the 80% a priori progression threshold.

Fidelity of assessment procedures followed agreed protocols. Fidelity measures indicated that the core components of strength and health education were delivered as intended. More sessions of cardiorespiratory fitness were delivered than intended and fewer sessions of flexibility, balance and mindfulness than planned were delivered, reflecting moderate fidelity (Table 5).

### 3.2 Physical Health Outcomes

This cohort was found to fit the category of Obese Class 1 at baseline (Purnell 2023) and with cardiorespiratory fitness below values previously reported within an intellectual disability's population (Guerra‐Balic et al. 2015), suggesting that this sample had a comparatively lower functional exercise capacity (Nasuti et al. 2013). There was a significant decrease in systolic blood pressure, and an increase in flexibility and lower body muscular endurance over the 8‐week period. Physical outcome results are presented in Table 6. Exploratory analyses of preliminary effectiveness by level of intellectual disability are shown in Table 7.

**TABLE 6:** Physical outcome measures at pre‐ and post‐intervention (*n* = 24).

Outcome measure	Pre (mean ± SD)	Post (mean ± SD)	Test of difference *p* value*	Effect size
Body weight (kg)	78.6 ± 19.9	78.4 ± 20.0	0.637	*d* = 0.10 (small)
BMI (kg/m^2^)	30 ± 6.7	30 ± 6.8	0.599	*d* = 0.11 (small)
Systolic blood pressure (mmHg)	120.8 ± 18.6	114.0 ± 15.7	**0.049**	*d* = 0.42 (medium)
Diastolic blood pressure (mmHg)	69.2 ± 9.8	67.2 ± 8.1	0.094	*d* = 0.36 (medium)
Lower body muscular endurance (reps)	12.6 ± 3.6	13.6 ± 3.5	**0.015**	*d* = 0.54 (medium)
Isometric handgrip strength (kg)	15.5 ± 7.3	15.0 ± 7.5	0.597	*d* = 0.11 (small)
Flexibility (cm)	34.0 ± 21.3	42.7 ± 19.1	**0.010**	*d* = 0.60 (medium)
Balance (s)	10.4 ± 16.7	12.1 ± 19.0	0.102	*d* = 0.38 (medium)
Cardiorespiratory fitness (m)	401 ± 74.0	414 ± 58.0	0.198	*d* = 0.27 (small)

*Note:* *p ≤ 0.05 indicates a significant difference. Results are for paired samples *t*‐tests. *d =* Cohen's *d*. Bold values indicate results that reached statistical significance.

**TABLE 7:** Findings from exploratory analyses pre‐ and post‐intervention.

Subgroup (*n*)	Outcome measure	Pre (mean ± SD)	Post (mean ± SD)	Test of difference *p* value*	Effect size
Mild ID (*n* = 9)	Weight (kg)	85.0 ± 20.3	85.6 ± 21.4	1.000^α^	*r* = 0.00
	Systolic blood pressure (mmHg)	124.6 ± 18.0	125.0 ± 17.5	0.529	*d* = 0.22
	Diastolic blood pressure (mmHg)	74.0 ± 13.1	71.6 ± 8.5	0.260	*d* = 0.40
	Lower body muscular endurance (reps)	13.0 ± 3.7	13.8 ± 3.7	0.133	*d* = 0.56
	Isometric handgrip strength (kg)	17.7 ± 7.7	19.6 ± 7.2	**0.047**	*d* = 0.78
	Flexibility (cm)	31.2 ± 21.1	41.9 ± 19.3	0.079	*d* = 0.73
	Balance (s)	20.0 ± 24.7	24.9 ± 26.5	**0.011**	*d* = 1.10
	Cardiorespiratory fitness (m)	462.25 ± 41.0	469.4 ± 21.1	0.808	*d* = 0.08
Moderate ID (*n* = 15)	Weight (kg)	72.9 ± 17.3	72.7 ± 17.5	0.511	*d* = 0.17
	Systolic blood pressure (mmHg)	117.1 ± 18.3	108.5 ± 12.3	0.051	*d* = 0.55
	Diastolic blood pressure (mmHg)	66.5 ± 7.1	65.0 ± 7.5	0.245	*d* = 0.31
	Lower body muscular endurance (reps)	12.3 ± 3.6	13.5 ± 3.6	0.059	*d* = 0.53
	Isometric handgrip strength (kg)	14.1 ± 6.9	12.3 ± 6.5	0.094	*d* = 0.46
	Flexibility (cm)	35.8 ± 22.1	43.2 ± 19.8	0.076	*d* = 0.51
	Balance (s)	4.6 ± 4.0	4.6 ± 4.6	0.677^α^	*r* = 0.14
	Cardiorespiratory fitness (m)	367.4 ± 68.9	387.3 ± 51.9	0.199	*d* = 0.35

*Note:* Pre and post values are presented as mean ± S.D. *p ≤ 0.05 indicates a significant difference. Results are for paired sample *t*‐tests unless indicated as ^α^. ^α^Related‐samples Wilcoxon Signed Rank Test. *d =* Cohen's *d*; *r* = standardised effect size. Bold values indicate results that reached statistical significance.

#### 3.2.1 Anthropometry

No significant changes in body weight and BMI were observed during the study. Full results are presented in Tables 6 and 7.

#### 3.2.2 Systolic Blood Pressure

There was a significant reduction in systolic blood pressure (*t* (23) = 0.0277, *p* = 0.049), with a mean reduction of 7 mmHg (*d* = 0.42, medium effect). Participants with a moderate level of intellectual disabilities showed a moderate effect (*d* = 0.552).

#### 3.2.3 Diastolic Blood Pressure

Diastolic blood pressure showed no significant change (*t* (23) = 1.749, *p* = 0.094), though a 2 mmHg reduction was a moderate effect (*d* = 0.36). Medium effects were noted for mild (*d* = 0.40) and moderate (*d* = 0.31) intellectual disability groups.

#### 3.2.4 Lower Body Muscular Endurance

Muscular endurance improved significantly with a medium effect (*t* (23) = −0.212, *p* = 0.015, *d =* 0.54). The mild and moderate intellectual disability subgroups showed medium effects (*d* = 0.56 and *d* = 0.53, respectively).

#### 3.2.5 Isometric Handgrip Strength

No significant overall change was observed (*t* (23) = 0.479, *p* = 0.597, *d =* 0.11). Participants with mild intellectual disabilities improved significantly with a medium effect (*t* (8) = −2.341, *p* = 0.047, *d* = 0.78); the moderate subgroup showed a medium effect (*d* = 0.46).

#### 3.2.6 Flexibility

Flexibility significantly improved (*t* (21) = −2.833, *p* = 0.01, *d* = 0.60, medium). Two participants declined retesting. Subgroup analysis found non‐significant but medium effects for the moderate intellectual disabilities subgroup (*d* = 0.51).

#### 3.2.7 Balance

Five participants (four with moderate intellectual disabilities) were unable to complete testing. Among completers (*n* = 19), a medium effect was detected (*d* = 0.38, medium effect), though not significant (*p* = 0.102). Subgroup analysis found a significant improvement with a large effect for participants with mild intellectual disabilities (*t* (8) = −3.113, *p* = 0.009, *d* = 1.10).

#### 3.2.8 Cardiorespiratory Fitness

There was no significant change in the 6MWT for the cohort (*t* (23) = −1.326, *p* = 0.198, *d* = 0.27). Subgroup analysis found a medium effect for participants with moderate intellectual disabilities (*d* = 0.35).

### 3.3 Personal Wellbeing Outcome

On a 0–100 scale, there was a mean total score across all domains of 87 (±15) at baseline and 86 (±13) post‐intervention, with no significant change found. Responses consistently at the maximum were excluded prior to analysis (*n* = 7).

## 4 Discussion

The principal finding of this study was that the intervention was feasible, acceptable, and delivered with good fidelity within a community‐based day service context for adults with intellectual disabilities. The preliminary effectiveness findings further support these implementation outcomes, providing evidence that the intervention can produce meaningful changes when delivered as intended, and thereby strengthening the case for progression to the next stage of evaluation.

### 4.1 Feasibility, Acceptability and Fidelity

Overall findings were positive, with measurements meeting most of the a priori criteria for progression. The recruitment rate aligns with previous studies that sought informed consent directly from individuals with intellectual disabilities (van Schijndel‐Speet et al. 2013, 2017), suggesting that the recruitment methods were appropriate for the population in the applied setting. High compliance compared favourably with previous studies (Lally et al. 2022; Melville et al. 2015). The deliberate planning by the intervention development team prior to this study to overcome pre‐identified barriers to compliance identified in previous studies, such as transport, availability of resources, and venues (van Schijndel‐Speet et al. 2014; Markle‐Reid et al. 2016; Glowacki et al. 2019), may have contributed to good compliance.

The intervention was delivered within the existing day service structure, and the high compliance and completion rates observed exceeded previous benchmarks (Ali et al. 2021) and are consistent with literature indicating that interventions integrated into familiar settings and routines are more likely to be feasible and acceptable for this population (Westrop et al. 2024). Evidence suggests that programmes embedded into existing schedules can reduce disruption and support ongoing participation (Hassan et al. 2019). There was no formal appraisal as to how distinct this intervention was from the usual daily activities, but the findings suggest feasibility was enhanced by its alignment with existing routines. Although activities varied across the week, positive feasibility and acceptability outcomes suggest that variation was acceptable within the predictable structure of the day service routine. The visual and verbal schedules provided during transitions may have supported participants in anticipating upcoming activities. In this study, the intervention was incorporated into the day service timetable, which may have reduced barriers related to attendance and engagement. The group‐based format may also have contributed to the feasibility outcomes observed. Peer modelling and social interaction have been associated with increased motivation and participation in adults with intellectual disabilities (Hutzler and Korsensky 2010; van Schijndel‐Speet et al. 2014), features inherent within the delivery model. Favourable recruitment and completion rates may be considered indirect indicators of acceptability, consistent with the theoretical framework of acceptability (Sekhon et al. 2017). Acceptability was additionally supported by the absence of adverse events indicating that participants were able to engage safely and consistently with the intervention, an indicator previously used as a feasibility metric in a vulnerable population (Korman et al. 2020).

However, important measurement challenges were identified. Completion rates were lower than the a priori thresholds for progression for two of the eight outcome measures. The inability of some participants to complete the balance test suggests that standard measures may not fully accommodate the range of physical abilities present in this cohort, particularly among those with moderate intellectual disability, and similar difficulty was previously noted in those with severe intellectual disabilities (Hale et al. 2007). Though unreported, the finding suggests that the validation cohort may have been closer to the milder side of the spectrum of disability severity (Blomqvist et al. 2012). The PWI‐ID was limited by a ceiling effect, despite all participants passing the pre‐test screen. The pre‐test includes specific items designed to detect acquiescent responding, whereby agreement with clearly incorrect statements would indicate susceptibility to response bias. However, it was acknowledged by the authors that the pre‐test is a screening tool and not a comprehensive assessment of response bias; therefore, data cleaning was necessary. Acquiescent responding is a concern affecting 20%–30% of respondents with intellectual disabilities, compared to 3%–5% for the general population (McGillivray et al. 2009), possibly due to social desirability, power differentials, perceived threat, or unfamiliar environments (Cummins and Lau 2010). The findings of ceiling effects are consistent with previously reported challenges in administering self‐report wellbeing tools in intellectual disabilities populations (Salomon et al. 2023). While these response patterns may reflect acquiescence or ceiling effects, it is also possible that some participants genuinely perceived their wellbeing to be very high. The elevated baseline scores also raise questions regarding the congruence between the current sample and that of the validation study (McGillivray et al. 2009; Salomon et al. 2023). There is a paucity of research at present that has utilised the PWI‐ID; however, the feasibility of a quality‐of‐life tool in the intellectual disabilities population has reported that people with intellectual disabilities should be considered ‘unreliable witnesses’, and carers or researcher involvement will almost always be required (Russell et al. 2018). In contrast, this study prioritised self‐report, supported by one‐to‐one administration and comprehension checks to identify self‐report capabilities. As the results show, the checks alone were insufficient to diminish ceiling effects.

Content fidelity indicated that intervention sessions were delivered within the range of moderate to high fidelity, and outcome measures procedures were applied consistently. The measurement provides this study with methodological strength through the explicit fidelity assessment, often unreported in feasibility studies (Bellamy et al. 2020; Salomon et al. 2018; van Schijndel‐Speet et al. 2014).

### 4.2 Preliminary Effectiveness

Within the context of generally strong feasibility, acceptability, and fidelity, the preliminary effectiveness findings provide further insight into how and for whom the intervention may work. The study reports statistically significant and meaningful improvements in several physical health outcomes for the cohort, suggesting that the intervention can confer cardiovascular and musculoskeletal benefits for adults with mild and moderate intellectual disabilities.

Systolic blood pressure decreased significantly, as previously found in the general population (Cornelissen and Smart 2013) and in those with intellectual disabilities (Oviedo et al. 2014). Conversely, no changes were found for weight or cardiorespiratory fitness (Calders et al. 2011; Oviedo et al. 2014). Effective weight change interventions included a diet component (Calders et al. 2011; Guidetti et al. 2010; Oviedo et al. 2014) and exercise duration and intensity were greater in effective studies targeting fitness (Calders et al. 2011; Oviedo et al. 2014). It is argued, however that in this cohort, self‐selected intensity may have contributed to programme compliance (Mehringer et al. 2025) but did not increase cardiorespiratory fitness in line with cardiovascular and strength improvements, despite the content fidelity assessment showing more cardiorespiratory training was delivered than planned. Flexibility improved across all participants, consistent with previous yoga‐based interventions, likely reflecting a sufficient dose–response (Csala et al. 2021; Reina et al. 2020) but additional strength and balance benefits were reported from yoga‐based intervention, suggesting training modalities confer multiple benefits (Reina et al. 2020).

Sensory, cognitive, or musculoskeletal factors, such as altered depth perception and joint instability, may contribute to the subgroup differences reported (Duplanty et al. 2014; Leyssens et al. 2022; Maïano et al. 2019). Handgrip strength, a recognised biomarker of health and mortality risk (Soysal et al. 2021; Vaishya et al. 2024), improved significantly in those with mild intellectual disabilities, which aligns with known associations between cognitive functioning and upper limb strength (Karatrantou et al. 2020). Balance improved only in participants with mild intellectual disabilities, contrasting with previous studies (Carmeli et al. 2005; Oviedo et al. 2014), and may potentially reflect a dose–response effect based on the content fidelity findings. The subgroup findings should be considered from a feasibility perspective. It is possible that the intervention as delivered was calibrated more appropriately for adults with mild intellectual disabilities. Further adaptation may be necessary for adults with moderate intellectual disabilities to fully benefit from the programme and outcomes. As such, the effectiveness differences noted by level of intellectual disability may reflect not only intervention effects, but also variation in the intervention and measurement fit across participants.

No significant changes were observed in overall personal wellbeing. PWI‐ID total scores exceeded Western normative data scores for the general population (McGillivray et al. 2009) and those reported in prior research involving adults with intellectual disabilities (Randall et al. 2023). This elevated baseline may have limited the potential for measurable improvement, complicating interpretation (Cummins and Lau 2010). Taken with the lower feasibility of the PWI‐ID reported, and the scant literature available utilising the scale (Lam et al. 2010; Randall et al. 2023), future trials should consider alternative instruments that are less susceptible to ceiling effects.

### 4.3 Strengths and Limitations

The health education component was well attended, but the absence of a formal assessment of learning limits interpretation of its impact. Future studies should use adapted materials, and accessible health literacy measures co‐developed with stakeholders.

The application of a priori thresholds was a notable strength, providing a structured and transparent framework for determining feasibility and guiding progression decisions consistent with the CONSORT extension (Eldridge et al. 2016). Although the sample was small, preliminary effectiveness across multiple outcomes suggests the intervention warrants testing in a larger, adequately powered trial. Reporting both statistical significance and effect sizes enhanced interpretability and supported assessment of feasibility and preliminary effectiveness given the sample size.

The feasibility evaluation identified outcome measures that were practical and acceptable for this population, whilst highlighting those that suggested burden or challenged self‐efficacy leading to withdrawal (Sekhon et al. 2017), particularly for those with moderate intellectual disabilities.

A further limitation was that the lead facilitator who delivered the intervention also conducted the baseline and post‐intervention assessments. Although fidelity with protocol was observed, this may have introduced assessor bias. Participants may also have felt motivated to provide favourable responses or perform optimally during physical assessments due to social desirability, potentially influencing outcomes (Cummins and Lau 2010).

Individuals with severe or profound intellectual disabilities were not represented in the study sample, limiting generalisability. While challenges of sampling and blinding in this population are well recognised (Oviedo et al. 2014), incorporating a control design and expanding inclusion criteria in future randomised trials would strengthen reach and internal validity.

A key limitation was that no data was collected on staff or participant experiences of the intervention. Future research should incorporate process evaluation to explore mechanisms driving change, stakeholder perspectives and contextual influences (Nordstrøm et al. 2013; Skivington et al. 2021). Given the role of the day service staff in supporting attendance, engagement and routine integration, future studies should also examine staff perspectives on feasibility and acceptability, and the supports required to facilitate intervention delivery and maintenance. Although progression of intervention gains to the home environment was beyond the scope of this study, future studies should examine the preconditions and supports necessary for this. Behaviours developed within structured settings may not readily generalise across contexts (Temple et al. 2017), and behavioural frameworks may help identify strategies to support sustained behaviour change (Michie et al. 2014).

Feasibility does not necessarily guarantee long‐term sustainability. Follow‐up assessments are needed to assess maintenance of effects to inform sustained behaviour change (Willems et al. 2022). This aligns with the MRC Framework emphasis on *how* and *why* interventions work, not just whether they do (Skivington et al. 2021).

## 5 Conclusion

Overall, the findings suggest that this multi‐component intervention was feasible, acceptable, and delivered with fidelity within a day service context for adults with intellectual disabilities. The observed feasibility and high engagement were consistent with evidence that interventions integrated into familiar environments and routines are more likely to be successfully implemented in this population (Hassan et al. 2019; Westrop et al. 2024). Preliminary effectiveness indicates that meaningful physical health improvements are achievable when the intervention is delivered as intended and supported appropriately. The findings highlight areas for refinement to inform future feasibility testing and progression to a randomised control trial.

## Funding

This project was funded by the No Barriers Foundation, Ireland.

## Ethics Statement

Ethics approval was obtained from the Research Ethics Committee (REC) of Atlantic Technological University (ATU), Donegal, Ireland (IREC‐ATUD‐23‐032). The study conforms with the ATU Research Ethics Policy (AQAE036) and the Declaration of Helsinki (World Medical Association, 2024).

## Consent

All participants provided their informed consent prior to their inclusion in the study. The study maintained the principles of the Declaration of Helsinki.

## Conflicts of Interest

This study is part of the author's PhD studies, which were funded by the No Barriers Foundation. No Barriers Foundation designed the programme evaluated, and a representative of the Foundation contributed to data collection (see acknowledgments). The funder was not involved in the conduct of the study beyond programme development and did not influence the analysis or reporting of the findings.

## Data Availability

The data that support the findings of this study are available on reasonable request from the corresponding author. The data are not publicly available due to privacy or ethical restrictions.
